# Evaluation of the In Vitro Antiparasitic Effect of the Essential Oil of *Cymbopogon winterianus* and Its Chemical Composition Analysis

**DOI:** 10.3390/molecules27092753

**Published:** 2022-04-25

**Authors:** Pedro Silvino Pereira, Carlos Vinicius Barros Oliveira, Ana Josicleide Maia, Maria Celeste Vega-Gomez, Miriam Rolón, Cathia Coronel, Antônia Eliene Duarte, Henrique Douglas Melo Coutinho, Abolghasem Siyadatpanah, Roghayeh Norouzi, Seyed Jafar Adnani Sadati, Polrat Wilairatana, Teresinha Gonçalves Silva

**Affiliations:** 1 Department of Antibiotics, Federal University of Pernambuco (UFPE), Av. Artur de Sá, s/n, Cidade Universitária, Recife 54740-520, PE, Brazil; teresinha100@gmail.com; 2 Laboratory of Pharmacology and Molecular Chemistry, Regional University of Cariri (URCA), 1161 Cel. Antonio Luiz Avenue, Crato 63105-000, CE, Brazil; viniciusbluesky@gmail.com (C.V.B.O.); anajosicleide.maia@gmail.com (A.J.M.); duarte105@yahoo.com.br (A.E.D.); 3 Centro Para El Desarrollo De La Investigación Científica (CEDIC), Fundación Moisés Bertoni, Manduvira 635, Asunción C.P. 1255, Paraguay; mcvegagomez@gmail.com (M.C.V.-G.); rolonmiriam@gmail.com (M.R.); cathiacoronel@gmail.com (C.C.); 4 Microbiology and Molecular Biology Laboratory, Regional University of Cariri (URCA), 1161 Cel. Antonio Luiz Avenue, Crato 63105-000, CE, Brazil; 5 Ferdows School of Paramedical and Health, Birjand University of Medical Sciences, Birjand 9717853577, Iran; asiyadatpanah@yahoo.com; 6 Department of Pathobiology, Faculty of Veterinary Medicine, University of Tabriz, Tabriz 516661647, Iran; roghayehnorouzi123@gmail.com; 7 Department of Microbiology & Immunology, Faculty of Medicine, Qom University of Medical Sciences, Qom 3736175513, Iran; jafaradnani@yahoo.com; 8 Department of Clinical Tropical Medicine, Faculty of Tropical Medicine, Mahidol University, Bangkok 10400, Thailand

**Keywords:** *C. winterianus*, geraniol, cytotoxicity, leishmanicidal, trypanocidal

## Abstract

*Cymbopogon winterianus*, known as “citronella grass”, is an important aromatic and medicinal tropical herbaceous plant. The essential oil of *C. winterianus* (EOCw) is popularly used to play an important role in improving human health due to its potential as a bioactive component. The present study aimed to identify the components of the essential oil of *C. winterianus* and verify its leishmanicidal and trypanocidal potential, as well as the cytotoxicity in mammalian cells, in vitro. The EOCw had geraniol (42.13%), citronellal (17.31%), and citronellol (16.91%) as major constituents. The essential oil only exhibited significant cytotoxicity in mammalian fibroblasts at concentrations greater than 250 μg/mL, while regarding antipromastigote and antiepimastigote activities, they presented values considered clinically relevant, since both had LC_50_ < 62.5 μg/mL. It can be concluded that this is a pioneer study on the potential of the essential oil of *C. winterianus* and its use against the parasites *T. cruzi* and *L. brasiliensis*, and its importance is also based on this fact. Additionally, according to the results, *C. winterianus* was effective in presenting values of clinical relevance and low toxicity and, therefore, an indicator of popular use.

## 1. Introduction

Neglected tropical diseases (NTDs) are a diverse group of tropical infections common in low-income populations in developing regions of Africa, Asia, and the Americas. Two of the main pathologies that fall into this category are *Mucocutaneous leishmaniasis* and *Trypanosomiasis*, caused by the protozoa *Leishmania braziliensis* and *Trypanosoma cruzi*, respectively, both belonging to the Trypanosomatidae family, class Kinetoplastea. Treatments for such infections are expensive, so it has been estimated that controlling NTDs would require between USD 2 billion and USD 3 billion in funding over the next five-to-seven years [1,2].

*Mucocutaneous leishmaniasis*, caused by the parasite *L. braziliensis*, leads to the partial or total destruction of the mucous membranes of the nose, mouth, and throat. More than 90% of cases of *Mucocutaneous leishmaniasis* occur in Bolivia, Brazil, Ethiopia, and Peru [3]. The use of chemotherapeutics, such as miltelfosine, and pentavalent antimonials, such as *N*-methyl-glucamine antimoniate, is often the first step in the treatment of Leishmania infections [4]. Recent evaluations have shown increased resistance to antimonials in some endemic areas, limiting their effectiveness and demanding a more species-specific solution when possible [5]. Two pentavalent antimony products available in the US are sodium stibogluconate and meglumine antimoniate. The recommended dosage for both is 20 mg/kg/day for 28 to 30 days in mucocutaneous and visceral leishmaniasis [6].

Chagas disease is caused by the protozoan *T. cruzi* transmitted by the feces of triatomines, which are contaminated with metacyclic trypomastigotes and ejected by the insect after feeding [7]. *T. cruzi* invades and multiplies as amastigotes within many different types of host cells, including muscle cells, macrophages, and fibroblasts [8]. Chagas disease is common in parts of Mexico, Central America, and South America, where an estimated 8 million people are infected [9]. Current options for the treatment of Chagas disease are restricted to benznidazole and nifurtimox, which are not well tolerated and imply frequent discontinuation of treatment [10].

In this scenario, there are essential oils (EOs) from several plant species with antiprotozoal properties that have become potential chemotherapeutic agents [11,12]. EOs are insoluble in inorganic solvents (water) but soluble in organic solvents (ether, alcohol, fixed oils). They are volatile liquids, with a characteristic odor, and are widely used in the perfumery, aromatherapy, and cosmetics industry; they are also increasingly important in the pharmaceutical scene due to their wide range of applications and biological activities [13]. 

Java citronella (*Cymbopogon winterianus* Jowitt ex Bor.) is an aromatic grass belonging to the Poaceae family that provides essential oils by stem distillation. It is widely used as a source in perfumery, soap, cosmetics, and flavoring industries. Leaf-blades are linear, tapering gradually to a long, membranous, acuminate shape, and up to 1 m long and 1.5 cm wide. *C. winterianus* is an industrially important perennial multi-crop cultivated in parts of tropical and subtropical areas of Asia, Africa, and America [14,15].

The study of *C. winterianus* in this research is due to its varied popular use and its diverse biological activities. Therefore, the present study aimed to identify the components and verify the leishmanicidal and trypanocidal power, as well as the in vitro cytotoxicity of the essential oil *C. winterianus* (EOCw).

## 2. Results

**Figure 1:**
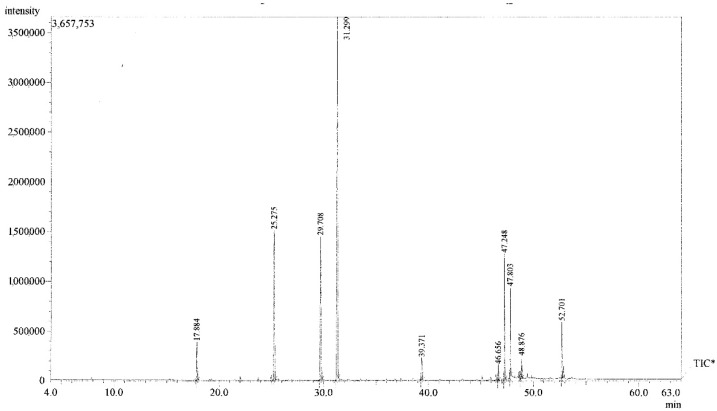
GC/MS chromatogram of the essential oil of *C. winterianus* with total ion current (TIC) peak reports. * Total Íon Current.

The chromatographic profile (chromatogram) of EOCw (Figure 1) revealed by gas chromatography with mass spectrometry (GC/MS), indicates a single substance with retention time = 31.299 min and maximum detection intensity (most concentrated). As can be seen in Table 1, the chemical composition of the EOCw presented as major constituents in ascending order of concentration: citronellol (16.91%), citronellal (17.31%), and geraniol (42.13%), followed by other compounds in lower concentrations.

**Figure 2:**
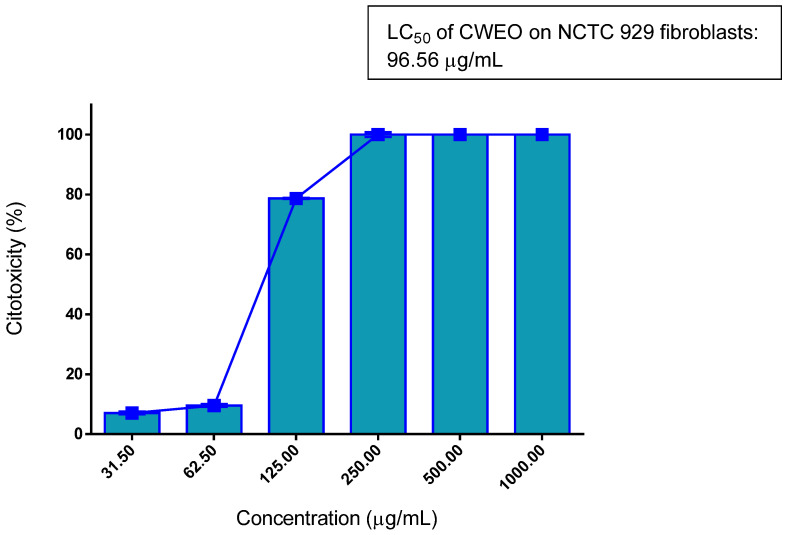
Cytotoxicity of the essential oil of *C. winterianus*, with the confidence interval for oil at 95% (87.07–107.10). LC_50_ was obtained through non-linear regression of means.

In Figure 2, it is graphically observed that the cytotoxic activity of the EOCw had a relatively high LC_50_ of 96.56 μg/mL, which is desired for products intended for human consumption.

Table 2 shows, from a different perspective, the same promising result observed in Figure 2, which would be the low cytotoxicity exhibited by EOCw against mammalian cells that would normally be infected by the parasites; cell damage only showed to be significant from the concentration of 125 µg/mL.

**Figure 3:**
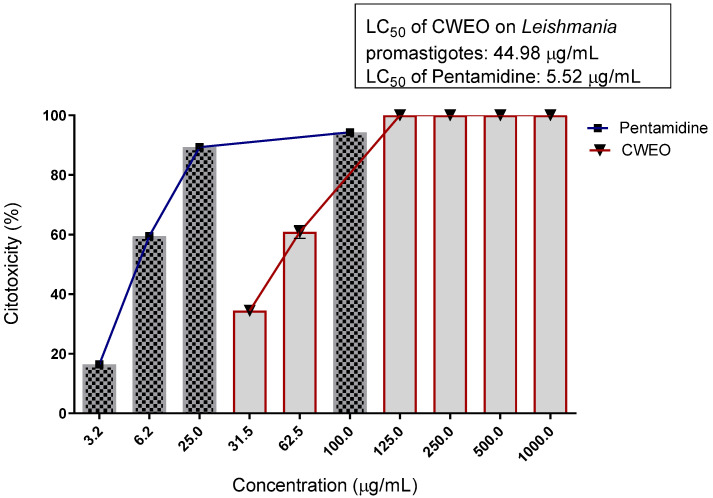
Cytotoxicity of the promastigote *L. brasiliensis* treated with essential oil of *C. winterianus.* LC_50_, with the confidence interval for oil at 95% (35.55–56.92). LC_50_ was obtained through non-linear regression of means.

**Figure 4:**
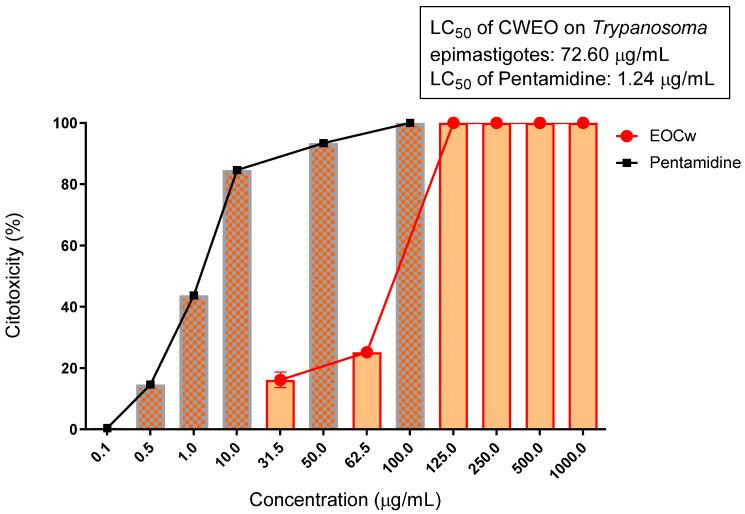
Cytotoxicity of the epimastigote *T. cruzi* treated with essential oil of *C. winterianus*. LC_50_ confidence interval for oil is 95% (50.63–104.10). LC_50_ was obtained through non-linear regression of means.

According to Figure 3 and Figure 4, the EOCw presented inhibition values considered very promising for the promastigote and epimastigote forms of *L. brasiliense* and *T. cruzi*, with LC_50_ values of 44.98 μg/mL and 72.60 μg/mL, for *Leishmania* and *Trypanosoma*, respectively, with performance equivalent to that of Pentamidine, at a concentration of 125 μg/mL.

Additionally, observing Table 3 and Table 4, it can be inferred that the EOCw showed survival percentage values considered very promising for the promastigote and epimastigote forms of *L. brasiliense* and *T. cruzi*, respectively.

**Figure 5:**
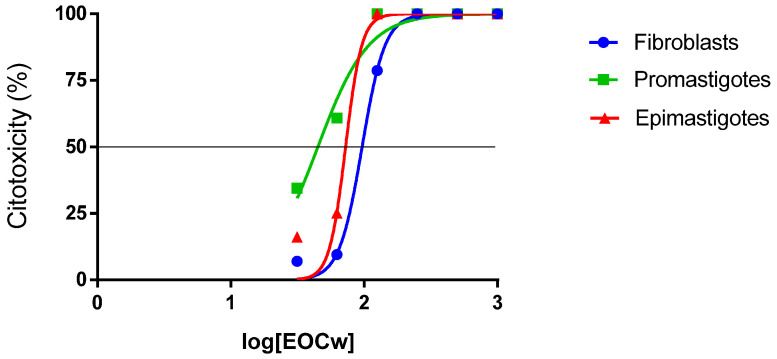
Log-dose vs. cytotoxicity curves for NCTC 929 fibroblasts, *L. braziliensis* promastigotes, and *T. cruzi* epimastigotes were estimated by non-linear regression of means.

Figure 5 shows the log-dose vs. cytotoxicity curves of EOCw for fibroblasts, promastigotes, and epimastigotes estimated through non-linear regression of means, demonstrating that the anti-kinetoplastid effect was more intense than the cytotoxic effect in fibroblasts.

## 3. Discussion

More than 200 different components present in pure essential oils have been reported. These mixtures typically contain phenylpropanoic derivatives or terpenes [16]. In general, gas chromatography (GC) is used for the analysis of volatile constituents present in essential oils, and liquid chromatography (LC) is used for the analysis of non-volatile constituents [17]. Usually, most essential oils are composed of volatile fractions, which contain monoterpenes, sesquiterpenes, and their oxygenated derivatives, where aliphatic alcohols, esters, and aldehydes may also be present [18,19].

The main compound found in the essential oil of *C. winterianus* using the GC technique with mass spectrometry was geraniol (3,7-dimethyl-2,6-octadien-8-ol) (42.13%), an acyclic monoterpenic alcohol in which biosynthesis occurs mainly in the cytosol of glandular trichomes via geranyl monophosphate (GP) through the action of a Nudix hydrolase [20,21]. Secondarily, citronellal (17.31%) and citronellol (16.91%) were detected in median concentrations, in which biosynthesis is related to the expression of heterodimeric geranyl diphosphate synthases (GPPS-SSU) and plastid geranylgeranyl diphosphate (GGPP) [22].

Essential oils of *Cymbopogon* spp. are diverse in chemical composition and have many bioactivities and potentials of great pharmaceutical and medicinal significance [23]. In this genus, it is possible to observe a remarkable variation in the composition and yield of the essential oil, which varies from 0.3% in *C. travancorensis* to 1.2% in *C. martinii*. The main components of citral essential oil “a” and “b” were detected in *Cymbopogon pendulus*, *C. flexuosus*, and *C. citratus* with the largest in *C. citratus*. *Cymbopogon confertiflorus* and *C. nardus* var. *confertiflorus* present an essential oil with a high content of geraniol (67.7% and 46.0%, respectively), and another group including *C. nardus* var. *nardus*, *C. nardus* var. *Java II*, and *C. winterianus* have less geraniol in their essential oil (ranging from 20% to 25%) [24,25].

Regarding the composition of the EOCw, the results obtained in the present study differ slightly from those already observed in the literature, according to which the proportion of citronellal (up to 40.23%) was higher than that of geraniol (up to 22.78%), with citronellol always having the third-highest proportion [26,27]. In the study by Kakaraparthi et al. [28], it is possible to observe that the proportion of geraniol has a significant positive correlation (0.60) with the maximum temperature to which the plant is exposed.

Studies that evaluated the cytotoxic effect of essential oils of species of the *Cymbopogon* genus on mammalian cells showed that these are usually similar to that observed in the present study for *C. winterianus* (LC_50_ = 96.56 µL/mL) (Figure 2 and Figure 5, Table 2) so that the LC_50_ varies in the range of 50–300 µL/mL, where an LC_50_ > 50 µL/mL is indicated as non-cytotoxic [29,30]. In fact, in *Cymbopogon* spp. with a similar proportion of constituents to that observed here in EOCW, a cytoprotective effect is observed [31]. In the literature, a great variation in the antioxidant capacity of EOCw can be observed (IC50 = 12. 66 ± 0.56/743 ± 18 μg/mL), especially with regard to its capacity to reduce the 2,2-diphenyl radical -1-picrylhydrazyl (DPPH), which may be related to its great variability in its composition [32,33].

Essential oil of *C. winterianus*, as well as that of *C. citratus*, has already been shown to inhibit cytotoxicity in murine neutrophils through a mechanism not related to free radical scavenging [34]. The low cytotoxicity observed here (Figure 2 and Figure 5, Table 2) is probably due to the absence of compounds known to be toxic to mammalian cells in EOCw (Table 1), such as citral [35]. In the study by Sinha et al. [36], a weak cytotoxic effect of citronella essential oil on human lymphocytes was observed, although the use of geraniol had the opposite effect.

Many human cell lines did not show genotoxic or clastogenic/aneugenic effects when exposed to high concentrations (100 μg/mL) of geraniol [37]. It has been shown that geraniol, despite its mild cytotoxicity in human lymphocytes, is able to scavenge free radicals similarly to butylated hydroxytoluene (BHT), ascorbic acid, and α-tocopherol, which are potent antioxidants [38]. Regarding citronellal, the second most-abundant compound in EOCw (Table 1), it is known to be non-toxic to mammalian cells (LC_50_ > 50 μg/mL) [30].

Few studies reporting the leishmanicidal effects of essential oils from species of the *Cymbopogon* genus have been reported, the majority of which cite the oil of *C. citratus* as a potent leishmanicidal agent, with LC50 values = 25 μg/mL and 1.7 µg/mL for *Leishmania infantum* and *L. amazonensis*, respectively, where the latter was superior to the pentamidine drug used here [39,40]. The leishmanicidal effects of EOCw observed here (Figure 3 and Figure 5, Table 3) are almost certainly due to a large number of oxygenated terpenes present in its composition (Table 1), which, as in *C. citratus*, demonstrate a strong leishmanicidal effect, especially for *L. braziliensis* [41].

Geraniol, identified here in large quantities in the EOCw (Table 1), did not show antipromastigote activity against *L. braziliensis* at a concentration of 100 µg/mL in the study by Carneiro et al. [42]; however, in the same study, it was shown that citronellal has excellent antipromastigote activity. Other plant species whose essential oils contain major amounts of citronellal, such as *Eucalyptus citriodora*, also exhibit good leishmanicidal effects [43]. Different species of *Leishmania* seem to be significantly affected by geraniol, which, in these cases, has LC_50_ = 3.78 µg/mL and 5.57 µg/mL for *L. infantum* and *L. major*, respectively [44].

In vitro analyses using molecular docking demonstrated that geraniol is strongly anchored by the molecules of *L. major* uridine diphosphate–glucose pyrophosphorylase (LmajUGPase), *L. major* methionyl t-RNA synthetase (LmajMetRS), and *L. infantum* nicotinamidase (LinfPnC1) [45]. The same type of evaluation indicated the spermidine synthase (SpdS) enzyme as a possible anchoring site for geraniol in *L. donovani*; however, linalool, with similar potential, does not present a good leishmanicidal effect [46,47]. Citral, molecularly similar to geraniol, is capable of causing considerable ultrastructural changes in *Leishmania* spp., including mitochondrial and kinetoplast swelling, autophagosomal structures, nuclear membrane rupture, and nuclear chromatin condensation, which would justify the antipromastigote effect observed here (Figure 3 and Figure 5, Table 3) [39]. Exposure to high concentrations of eugenol, another oxygenated monoterpene, also caused extensive fatal cell damage in *Leishmania* spp. promastigotes, such as cells with two or more flagella, swollen mitochondria and altered inner mitochondrial membrane, with a significant increase in the number of cristae, indicating an associated mechanism of action. to mitochondrial damage [48].

The performance of EOCw in inhibiting the epimastigote form of *Trypanosoma cruzi* (Figure 4 and Figure 5, Table 4) was similar to that observed in studies of the same scope using species of the genus *Cymbopogon* [49,50]. *Cymbopogon citratus* has already demonstrated a potent trypanocidal effect (LC_50_ = 3.2 μg/mL) against *Trypanosoma brucei*, that is, almost as efficient as the standard drug pentamidine used in our study, where its major component, citral, was found to be responsible for this performance, presenting a similar effect (LC_50_ = 18.9 μg/mL) [51].

Different forms of *T. cruzi* have already been shown to have different sensitivities to oxygenated monoterpenes such as geraniol and citronellal; trypomastigote forms are much more susceptible to its cytotoxic effects than epimastigotes [52]. Citral, abundant in *Cymbopogon citratus* essential oil, exhibits an exceptional trypanocidal effect on epimastigotes, presenting an LC_50_ = 42 μg/mL for *T. cruzi*, which is not observed in different preparations such as methanol extracts (68.25 μg/mL), probably due to the low concentration of citral [53,54,55].

One study observed that the antiproliferative effect of *C. citratus* essential oil was derived from its main constituent (citral) in the three evolutionary forms of *T. cruzi* (LC_50_/24 h < 50 μg/mL for citral), which, based on the ultrastructural analysis, induce cytoplasmic and nuclear extraction, while the plasma membrane remained morphologically preserved in the parasites [56]. *T. cruzi* cells exposed to oxygenated monoterpenes such as geraniol and citral showed typical characteristics of apoptosis, such as cytoplasmic bubble, cell shrinkage, absence of flagellum, loss of mitochondrial membrane potential, nuclear chromatin condensation, and DNA fragmentation probably due to loss of mitochondrial function [57,58].

## 4. Materials and Methods

### 4.1. Plant Material, Selection, and Identification

Cymbopogon winterianus leaves were collected in the morning at the Medicinal Plants Garden of the Regional University of Cariri (URCA), Crato, Ceará, Brazil, and authenticated by Prof. Afranio Fernandes at the Department of Biology at the Federal University of Ceará. Specimens of the plant are deposited at the Herbarium Prisco Bezerra, Fortaleza, Ceará, Brazil, voucher n° 43.194. 

### 4.2. Obtaining Essential Oil from C. winterianus

Fresh leaves were cut into pieces and then washed and macerated with 99.9% ethanol for 72 h at room temperature. The essential oil was obtained in a Clevenger apparatus by hydrodistillation. Fresh leaves of *C. winterianus* were placed in a 5 L flask, together with 3 L of distilled water, and heated for 2 h. Then, the obtained mixture was separated, and the essential oil of *C. winterianus* was treated with anhydrous sodium sulfate, filtered, and kept under refrigeration until the moment of analysis.

### 4.3. Essential Oil Chemical Identification

The mass detection method applied in this study (secondary electron multiplier with conversion dynode) has the highest sensitivity and is the most suitable technique for estimating concentrations [59]. Oil analysis was performed with a Shimadzu GC/MS apparatus—QP2010 series (GC/MS system): Rtx-5MS capillary column (30 m × 0.25 mm, film thickness of 0.25 μm); helium carrier gas at 1.5 mL/min; injector temperature 250 °C; detector temperature 290 °C; column temperature 60–180 °C to 5 °C/min, then 180–280 °C to 10 °C/min (10 min). The scan speed was 0.5 scan/sec from m/z 40 to 350, and the split ratio was 1:200. Injected volume was 1 µL of (25 µL (essential oil)/5 mL CHCl_3_) (1:200). The solvent cut-off time was 2.5 min. The mass spectrometer was operated using 70 eV of ionization energy. The identification of the individual components was based on their mass spectral fragmentation based on the NIST 08 mass spectral library, retention indices, and comparison with published data. The relative retention rates were disregarded, since the manufacturer of the chromatography equipment used (Shimadzu^©^) indicated that measurement errors would increase for target peaks located far from the reference peak, making it difficult to find a relationship with a chemical structure [60].

### 4.4. Antiparasitic Activity

#### 4.4.1. Cell Lines Used 

Strains of CL-B5 parasites (clone CL-B5) were used for in vitro evaluation of activity on *T. cruzi* [61]. Parasites transfected with the β-galactosidase gene from *Escherichia coli* (LacZ) were provided by Dr. F. Buckner through the Gorgas Memorial Institute (Panamá). The epimastigote forms were cultivated in tryptose liver infusion at 28 °C, supplemented with 10% fetal bovine serum (FBS), 10 U/mL of penicillin, and 10 μg/mL of streptomycin at pH 7.2 and incubated with different concentrations of essential oil (1000; 500; 250; 125; 62.5; 31.5 μg/mL) and collected during the exponential growth phase [62].

The in vitro antileishmanial activity was established using *L. braziliensis* promastigotes (MHOM/CW/88/UA301) at 26 °C, cultured in Schneider’s medium for insects, supplemented with 10% (*v/v*) of fetal serum heat-inactivated calf, 2% normal human urine (*v/v*) plus penicillin and streptomycin [62]. The forms were seeded and incubated with different concentrations of essential oil (1000; 500; 250; 125; 62.5; 31.5 μg/mL).

#### 4.4.2. Reagents

Resazurin sodium substance was obtained from Sigma-Aldrich (St. Louis, MO, USA) and stored at 4 °C protected from light. Resazurin solution was prepared with 1% phosphate buffer, pH 7, and previously sterilized by filtration. Subsequently, Chlorophenol red-β-D-galactopyranoside (CPRG, Roche, Indianapolis, IN, USA) was dissolved in a 0.9% solution of Triton X-100 (pH 7.4). Penicillin G (Ern, SA, Barcelona, Spain), streptomycin (Reig Jofre SA, Barcelona, Spain), and dimethylsulfoxide (DMSO) were also used.

#### 4.4.3. In Vitro Epimastigote Sensitivity Assay

Assays were performed as described by Vega et al. [63], with crops that did not reach the stationary phase. Epimastigote forms were seeded at 1 × 10^5^ per mL in 200 μL, in 96-well microdilution plates, which were incubated at 28 °C for 72 h. Then, 50 µL of CPRG solution was added to give a final concentration of 200 µM. Plates were incubated at 37 °C for an additional 6 h. Absorbance reading was performed in a spectrophotometer at 595 nm. Concentrations were tested in triplicate. Each experiment was performed twice separately. The percentage of inhibition (% AE) was calculated as follows:

% AE = [(AE_AEB)/(AC_ACB)] × 100

where AE = absorbance of the experimental group; AEB = compound blank; AC = absorbance control group; CBA = culture environment blank. The essential oil was previously dissolved in DMSO. The concentration of DMSO (dimethylsulfoxide) used to allow the solubility of the oil was not greater than 0.01%. The effectiveness of Pentamidine as a trypanocidal drug was also determined.

#### 4.4.4. In Vitro Leishmanicidal Assay

The tests were performed according to Mikus and Steverding [64] with some modifications. Oil activity was evaluated in triplicate. Promastigote forms (2.5 × 10^5^ parasites/well) were cultured in 96-well plastic plates. Samples were dissolved in dimethylsulfoxide (DMSO). Different dilutions of compounds up to 200 mL of the final volume were added. After 48 h at 26 °C, 20 µL of resazurin solution was added and the oxidation-reduction was measured at 570 to 595 nm. Percentages of antipromastigotes (AP%) were calculated. The effectiveness of the reference leishmanicidal drug pentamidine was also determined.

### 4.5. Fibroblasts Cytotoxic Assays

To measure cell viability in mammalian cells, a colorimetric assay with resazurin was used. NCTC 929 fibroblasts were seeded (5 × 10^4^ cells/well) in 96-well flat-bottom microdilution plates with 100 µL of RPMI 1640 medium for 24 h at 37 °C and cultured in 5% CO_2_ for cells to adhere to the plates. The medium was replaced by different concentrations of essential oil (1000; 500; 250; 125; 62.5; 31.5 μg/mL) in 200 μL of medium and incubated for another 24 h. Growth controls were included. Then, a volume of 20 μL of 2 mM resazurin solution was added and the plates were placed in the incubator for another 3 h to assess cell viability. Resazurin reduction was determined by measuring the wavelength absorbance at 490 nm and 595 nm. Each concentration was tested three times. The cytotoxicity of each compound was estimated by calculating the percentage of cytotoxicity (%C).

### 4.6. Statistical Analysis

Results are expressed as mean ± standard error of the mean (SEM) of three independent experiments performed in triplicate. Concentrations capable of causing 50% lethality (LC_50_) were calculated by non-linear regression log-dose vs. mean ± standard error of the mean, using GraphPad Prism software version 6.0.

## 5. Conclusions

This study elucidated the composition and revealed, for the first time, the antiparasitic effect of the essential oil of *C. winterianus*, especially against *L. braziliensis*, but also against *T. cruzi*. Furthermore, we were able to determine a low cytotoxic effect of this natural product against mammalian cells, which enables possible applications in vivo. Therefore, future studies with the isolated constituents of the essential oil of *C. winterianus* need to be evaluated regarding the molecular mechanisms of interaction with kinetoplastid and human cells.

## Figures and Tables

**Table 1:** Chemical composition (%) of the essential oil of *C. winterianus*.

Components	RT (min) ^a^	(%)
Limonene	17.88	4.24
Citronellal	25.27	17.31
Citronellol	29.70	16.91
Geraniol	31.29	42.13
*β*-elemene	39.37	2.69
δ-Cadinene	46.65	1.05
Elemol	47.24	6.71
Germacrene	47.80	4.44
Guaiol	48.87	1.14
Nerolidol	52.70	3.38
Total		100.00

^a^ Retention time.

**Table 2:** Survival of fibroblasts exposed to the essential oil of *C. winterianus*.

Natural Product	Conc. µg/mL	%C	±%DS
*C. winterianus*	1000	0	–
	500	0	–
	250	2.15	0.49
	125	35.79	0.80
	62.5	89.96	0.70
	31.5	90.96	0.77

**Table 3:** Survival of the promastigote *L. brasiliensis* treated with the essential oil of *C. winterianus*.

Natural Product	Conc. µg/mL *C. winterianus*	%S	±%DS	Conc. µg/mL Pentamidine	%S	±%DS
*C. winterianus*	1000	0	-			
	500	0	-			
	250	0	-			
	125	0	-			
				100	5.7	0.2
	62.5	39.13	2.11			
	31.5	65.56	1.02			
				25	10.7	0.4
				6.2	40.5	0.2
				3.2	83.6	0.9

**Table 4:** Survival of the epimastigote *T. cruzi* treated with the essential oil of *C. winterianus*.

Natural Product	Conc. µg/mL *C. winterianus*	%S	± %DS	Conc. g/mL Pentamidine	%S	±%DS
*C. winterianus*	1000	0	–			
	500	0	–			
	250	0	–			
	125	0	–			
				100	0	0.7
	62.5	74.89	1.70			
				50	6.6	0.5
	31.5	83.88	2.52			
				10	15.4	0.6
				1.0	56.3	0.5
				0.5	85.4	0.6
				0.1	99.6	0.3

## Data Availability

Not applicable.

## Sample Availability

Samples of the compound are not available from the authors.
